# The complete mitochondrial DNA of the Cuban gar (*Atractosteus tristoechus*)

**DOI:** 10.1080/23802359.2017.1339214

**Published:** 2017-06-14

**Authors:** Gabriela Ulmo-Díaz, Andrés Hurtado, Jeremy Le Luyer, Erik García-Machado, Louis Bernatchez

**Affiliations:** a Centro de Investigaciones Marinas, Universidad de La Habana, La Habana, Cuba;; b Centro de Reproducción para la Ictiofauna Indígena, Matanzas, Cuba;; c Institut de Biologie Intégrative et des Systèmes (IBIS), Université Laval, Québec, Canada

**Keywords:** Endemic species, freshwater fish, Lepisosteidae, mitogenome, phylogeny

## Abstract

The Cuban gar (*Atractosteus tristoechus*) is an endemic lepisosteid living in Cuba. Among gars, this species is one of the most threatened and has the smallest natural distribution range. Lepisosteids are air-breathing fishes belonging to the Holostean, a basal non-teleost clade of actinopterygians. Recent studies have indicated that these fishes could be a ‘bridge between tetrapods and teleost biomedical models’. Herein, we sequenced and assembled the first complete mitochondrial genome of *A. tristoechus*. The total length of the mt genome is 16,290 bp, containing the typical 13 protein-coding genes, two ribosomal RNA (rRNA) genes, 22 transfer RNA (tRNA) genes, and a 537 bp length control region.

The Cuban gar (*Atractosteus tristoechus*) is an endemic lepisosteid living in marshes and rivers of southwestern Cuba. It has the smallest natural distribution among the members of the Lepisosteidae family. A recent study revealed very low levels of genetic diversity in the species (Ulmo-Díaz et al. 2016). Gars have been reported to share convergent genomic characteristics with mammals (Braasch et al. 2016; Symonová et al. 2016). Herein, we sequenced and assembled the first complete mitogenome of *A. tristoechus*.

A fin tissue sample from an adult female of *A. tristoechus,* caught at Zapata Swamp, Cuba and kept in captivity to be used as brood stock at the Center for Native Ichthyofauna Reproduction, was preserved in 96% ethanol and kept at 4 °C. Total DNA was extracted using a salt extraction protocol (Aljanabi and Martinez 1997) and stored at Bernatchez Lab. The DNA libraries were constructed with a NEBNext Ultra II DNA library preparation kit (New England Biolabs, Ipswich, MA) and run on Illumina MiSeq (paired-end 300 reads) at IBIS Genomics facility (Université Laval). The *de novo* assembly was carried out with A5-miseq pipeline (Coil et al. 2015). Sequences were prior trimmed for adaptors, minimum length and minimum quality with Trimmomatic v0.36 software (Bolger et al. 2014) fixing leading (3), trailing (3), sliding windows (4:15), and minlen (36) parameters. Assembly was further aligned and checked using the complete *Atractosteus spatula* mitogenome (Genbank accession number: AP004355.1) as reference, using MEGA7 (Kumar et al. 2016). Gene annotation was done using MitoAnnotator (Iwasaki et al. 2013) and MITOS (Bernt et al. 2013) software. Complete mitochondrial genome sequences of other five lepisosteid species were used for phylogenetic analysis. Additionally, sequences of *Amia calva* and *Danio rerio* were used as out groups. ClustalW was used for alignments using MEGA7. A maximum-likelihood phylogenetic tree was constructed using the GTR substitution model (Rodríguez et al. 1990) with gamma parameter *α* = 0.31, in MEGA7.

*Atractosteus tristoechus* complete mitogenome (GenBank accession number: KY581571) has 16,290 pb length and 44% GC content. The mitogenome structure includes 13 protein-coding genes, two rRNA genes (12S and 16S), 22 tRNA genes and a control region (D-loop, located between tRNA-Pro and tRNA-Phe). Main mitogenome features in terms of structure; GC content and gene order were similar to other gar species (Inoue et al. 2003; Broughton and Reneau 2006; Del Río-Portilla et al. 2016; Yu et al. 2016). *A. tristoechus* was phylogenetically close to *A. spatula* (Figure 1) as shown previously (Wright et al. 2012). A slow mitogenome mutation rate is one of the features highlighted in this family (Bernatchez and Wilson 1998; Rabosky et al. 2013). Notably, *Lepisosteus oculatus* and *Lepisosteus platyrhincus* mitogenomes show an extremely low genetic divergence between, with only three variable nucleotides (*d* = 0.0002 ± 0.00009) over the entire mitogenome. However, genetic distance between these species may vary depending on the geography of the populations sampled. Sipiorski (2011) reported that *L. oculatus* sampled from Apalachicola River, western Florida, was more closely related to *L. platyrhincus* than to *L. oculatus* from other geographically distant populations. This has been explained as result of introgressive hybridization, common in freshwater fish species (Hubbs 1955; April et al. 2011), including gars species (Herrington et al. 2008; Bohn et al. 2017).

**Figure 1.**
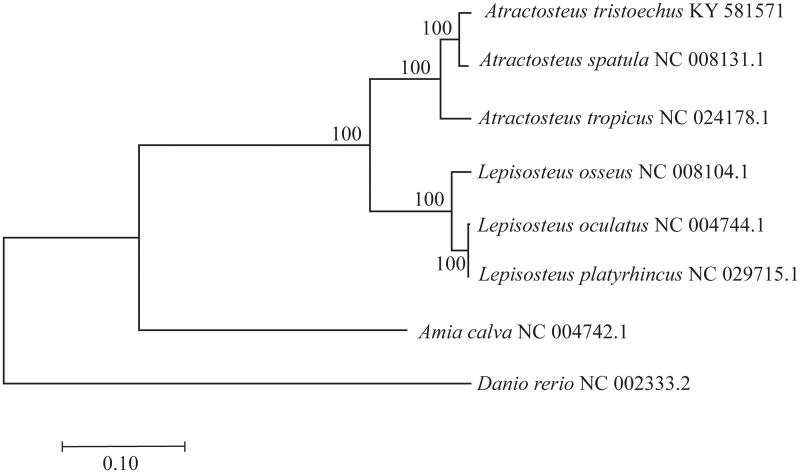
Full mtDNA maximum likelihood phylogenetic tree of six of the seven extant lepisosteids, obtained using the GTR + G substitution model. Values on nodes are bootstrap values (after 1000 replicates).

The information regarding the *A. tristoechus* mitogenome will contribute to the effort for the conservation of this endangered species.
